# 3-Phenyl-1-[2-(3-phenyl­isoquinolin-1-yl)­diselan­yl]isoquinoline

**DOI:** 10.1107/S160053680803609X

**Published:** 2008-11-08

**Authors:** Venkatesha R. Hathwar, K. Prabakaran, R. Subashini, P. Manivel, F. Nawaz Khan

**Affiliations:** a Solid State and Structural Chemistry Unit, Indian Institute of Science, Bangalore 560 012, Karnataka, India; b Organic Chemistry Division, School of Science and Humanities, VIT University, Vellore 632 014, Tamil Nadu, India

## Abstract

The complete molecule of the title compound, C_30_H_20_N_2_Se_2_, is generated by a crystallographic inversion centre at the mid-point of the Se—Se bond. The dihedral angle between the isoquinoline-1-selenol group and the phenyl ring is 14.92 (2)°. The herringbone-like packing of the structure is supported by inter­molecular π–π stacking inter­actions with a shortest perpendicular distance between isoquinoline groups of 3.514 Å; the slippage between these ring systems is 0.972 Å, and the distance between the centroids of the six-membered carbon rings is 3.645 (3) Å.

## Related literature

For biological properties of organoselenium compounds, see: Mugesh & Singh (2000 ▶). For chemopreventive agents in human cancer therapy, see: Sugie *et al.* (2000 ▶).
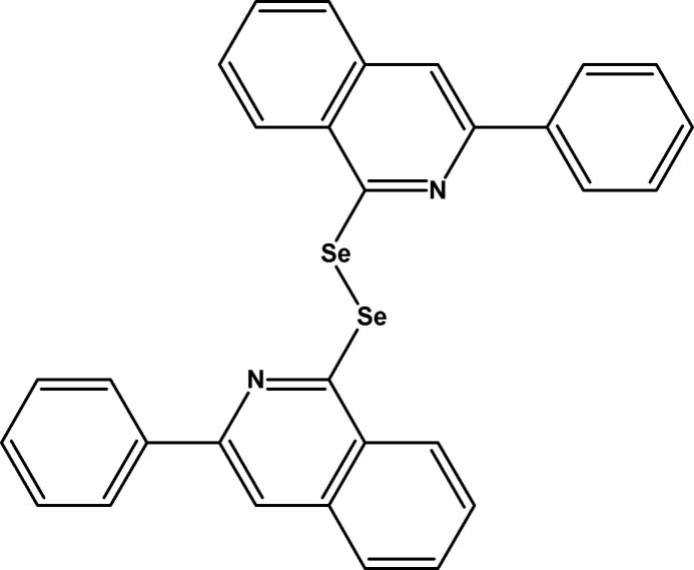


## Experimental

### 

#### Crystal data

C_30_H_20_N_2_Se_2_
*M* *_r_* = 566.40
Monoclinic, 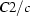

*a* = 11.2441 (17) Å
*b* = 17.559 (3) Å
*c* = 13.248 (3) Å
β = 115.082 (2)°
*V* = 2369.0 (8) Å^3^
*Z* = 4
Mo *K*α radiation
μ = 3.14 mm^−1^
*T* = 290 (2) K
0.20 × 0.14 × 0.11 mm

#### Data collection

Bruker SMART CCD area-detector diffractometer
Absorption correction: multi-scan (*SADABS*; Sheldrick, 1996 ▶) *T*_min_ = 0.585, *T*_max_ = 0.703
8668 measured reflections
2207 independent reflections
1516 reflections with *I* > 2σ(*I*)
*R*_int_ = 0.040

#### Refinement

*R*[*F* ^2^ > 2σ(*F* ^2^)] = 0.043
*wR*(*F* ^2^) = 0.102
*S* = 1.00
2207 reflections
158 parameters
H-atom parameters constrained
Δρ_max_ = 0.66 e Å^−3^
Δρ_min_ = −0.22 e Å^−3^

### 

Data collection: *SMART* (Bruker, 2004 ▶); cell refinement: *SAINT* (Bruker, 2004 ▶); data reduction: *SAINT*; program(s) used to solve structure: *SHELXS97* (Sheldrick, 2008 ▶); program(s) used to refine structure: *SHELXL97* (Sheldrick, 2008 ▶); molecular graphics: *ORTEP-3* (Farrugia, 1997 ▶) and *CAMERON* (Watkin *et al.*, 1993 ▶); software used to prepare material for publication: *PLATON* (Spek, 2003 ▶).

## Supplementary Material

Crystal structure: contains datablocks global, I. DOI: 10.1107/S160053680803609X/si2124sup1.cif

Structure factors: contains datablocks I. DOI: 10.1107/S160053680803609X/si2124Isup2.hkl

Additional supplementary materials:  crystallographic information; 3D view; checkCIF report

## Figures and Tables

**Fig. 1.**
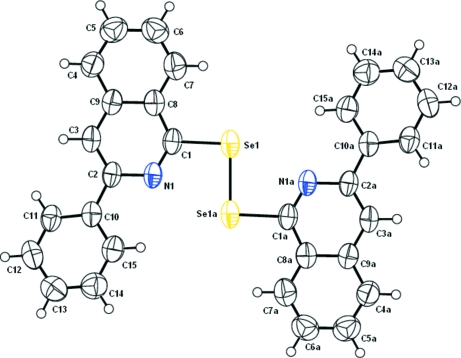
ORTEP diagram of molecule (I) with 50% probability displacement ellipsoids. The diselenide link is formed between Se1 and its symmetry equivalent at (3/4, 1/4, 1).

**Fig. 2.**
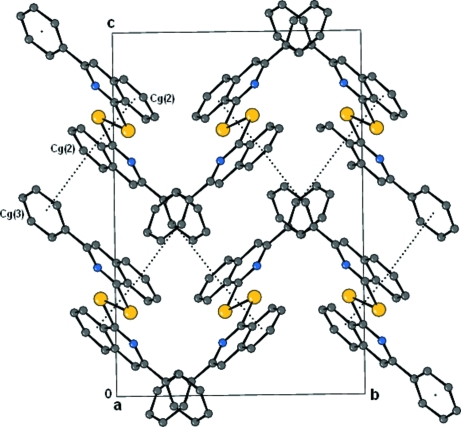
The crystal packing diagram of (I). The dotted lines indicate intermolecular π–π aromatic stacking interactions. All H atoms have been omitted for clarity. Cg2 and Cg3 are the centroids of the C4—C9 ring and C10—C15 ring, respectively.

## supplementary crystallographic information

### Comment

Organoselenium compounds are widely used in modern organic synthesis, materials synthesis, biochemistry, photography, ligand chemistry, electroconducting materials and biologically relevant properties like antibacterial, antiviral, antifungal, antiparastic and antiradiation (Mugesh & Singh, 2000 and references therein). Organoselenium compounds are less toxic and more chemopreventive in comparision with that of inorganoseleniums and natural organoseleniums. Hence, organoseleniums are considered as better candidates of chemopreventive agents for human cancers. (Sugie *et al.*, 2000).

The structure has one half-molecule in the asymmetric unit (*Z*' = 1/2) with the molecule sitting on a crystallographic inversion centre, which is located in the middle of the Se–Se bond. The title compound (I) was obtained by a diselenide link, which is formed between Se1 and its symmetry equivalent at (3/4, 1/4, 1) (Fig. 1). The angle between the isoquinoline-1-selenol moiety and the phenyl ring is 14.92 (2)° indicating that the phenyl ring is twisted with respect to the isoquinoline-1-selenol backbone.

The crystal packing diagram does not have any significant weak intermolecular interactions whereas the herringbone-like packing of the structure (Fig.2) is supported by intermolecular π···π [*Cg*2···*Cg*2^ii^ with the symmetry code ii = 5/2 - *x*, 1/2 - *y*, 2 - *z*.] stacking interactions with a shortest perpendicular distance between isochinoline groups of 3.514 Å, the slippage between these ring systems is 0.972 Å, the distance between the centroids of the six-membered carbon rings C4/C9 is 3.645 (3) Å. Similarly, another intermolecular π···π [*Cg*2···*Cg*3^iii^] stacking interaction with a shortest perpendicular distance of 3.768 Å between the two rings and the distance between the centroids of the six-membered carbon rings is 3.917 (3) Å with the symmetry code iii = 1-*x*,-y,-*z. Cg*2 and *Cg*3 are the centroids of C4/C9 ring and C10/C15 ring, respectively.

### Experimental

A mixture of 1-chloro-3-phenylisoquinoline (1 mmol) and selenourea (1.1 mmol) in ethanol was vigorously stirred at ambient temperature for 2 hr. After completion of the reaction as indicated by TLC, solvent was removed and the reaction mixture was poured into water (10 ml) and the product was extracted using ethyl acetate (3X10 ml). The combined ethyl acetate extracts were concentrated *in vacuo*. The resulting crude product was directly charged onto a small silica gel column and eluted with a mixture of ethyl acetate/petroleum ether to get the final product of the diselenide title compound. Brown crystals of (I) were recrystalized from ethylacetate.

### Refinement

All the H atoms in (I) were positioned geometrically and refined using a riding model with C—H = 0.93Å and *U*_iso_(H) = 1.2*U*_eq_(C) for aromatic H atoms.

### Crystal data

**Table:**

C_30_H_20_N_2_Se_2_	*F*_000_ = 1128
*M**_r_* = 566.40	*D*_x_ = 1.588 Mg m^−^^3^
Monoclinic, *C*2/*c*	Mo *K*α radiation λ = 0.71073 Å
Hall symbol: -C 2yc	Cell parameters from 948 reflections
*a* = 11.2441 (17) Å	θ = 2.3–24.6º
*b* = 17.559 (3) Å	µ = 3.14 mm^−^^1^
*c* = 13.248 (3) Å	*T* = 290 (2) K
β = 115.082 (2)º	Block, brown
*V* = 2369.0 (8) Å^3^	0.20 × 0.14 × 0.11 mm
*Z* = 4	

### Data collection

**Table:**

Bruker SMART CCD area-detector diffractometer	2207 independent reflections
Radiation source: fine-focus sealed tube	1516 reflections with *I* > 2σ(*I*)
Monochromator: graphite	*R*_int_ = 0.040
*T* = 290(2) K	θ_max_ = 25.5º
φ and ω scans	θ_min_ = 2.3º
Absorption correction: multi-scan(SADABS; Sheldrick, 1996)	*h* = −13→13
*T*_min_ = 0.585, *T*_max_ = 0.703	*k* = −21→19
8668 measured reflections	*l* = −16→16

### Refinement

**Table:**

Refinement on *F*^2^	Secondary atom site location: difference Fourier map
Least-squares matrix: full	Hydrogen site location: inferred from neighbouring sites
*R*[*F*^2^ > 2σ(*F*^2^)] = 0.043	H-atom parameters constrained
*wR*(*F*^2^) = 0.102	*w* = 1/[σ^2^(*F*_o_^2^) + (0.0594*P*)^2^] where *P* = (*F*_o_^2^ + 2*F*_c_^2^)/3
*S* = 1.00	(Δ/σ)_max_ < 0.001
2207 reflections	Δρ_max_ = 0.66 e Å^−^^3^
158 parameters	Δρ_min_ = −0.22 e Å^−^^3^
Primary atom site location: structure-invariant direct methods	Extinction correction: none

### Special details

**Table:**

Geometry. All e.s.d.'s (except the e.s.d. in the dihedral angle between two l.s. planes) are estimated using the full covariance matrix. The cell e.s.d.'s are taken into account individually in the estimation of e.s.d.'s in distances, angles and torsion angles; correlations between e.s.d.'s in cell parameters are only used when they are defined by crystal symmetry. An approximate (isotropic) treatment of cell e.s.d.'s is used for estimating e.s.d.'s involving l.s. planes.
Refinement. Refinement of *F*^2^ against ALL reflections. The weighted *R*-factor *wR* and goodness of fit *S* are based on *F*^2^, conventional *R*-factors *R* are based on *F*, with *F* set to zero for negative *F*^2^. The threshold expression of *F*^2^ > σ(*F*^2^) is used only for calculating *R*-factors(gt) *etc*. and is not relevant to the choice of reflections for refinement. *R*-factors based on *F*^2^ are statistically about twice as large as those based on *F*, and *R*-factors based on ALL data will be even larger.

### Fractional atomic coordinates and isotropic or equivalent isotropic displacement parameters (Å^2^)

**Table:**

	*x*	*y*	*z*	*U*_iso_*/*U*_eq_	
Se1	0.85678 (4)	0.23078 (2)	1.05937 (3)	0.0658 (2)	
N1	0.8556 (3)	0.35522 (16)	0.9293 (2)	0.0532 (7)	
C1	0.9346 (4)	0.3092 (2)	1.0051 (3)	0.0546 (9)	
C2	0.9077 (3)	0.41108 (19)	0.8880 (3)	0.0509 (9)	
C3	1.0405 (4)	0.4174 (2)	0.9231 (3)	0.0590 (10)	
H3	1.0741	0.4550	0.8929	0.071*	
C4	1.2651 (4)	0.3718 (3)	1.0436 (3)	0.0732 (12)	
H4	1.3018	0.4088	1.0153	0.088*	
C5	1.3442 (4)	0.3224 (3)	1.1219 (4)	0.0837 (13)	
H5	1.4347	0.3251	1.1456	0.100*	
C6	1.2919 (5)	0.2680 (3)	1.1666 (4)	0.0815 (13)	
H6	1.3474	0.2354	1.2218	0.098*	
C7	1.1602 (5)	0.2619 (2)	1.1307 (3)	0.0745 (12)	
H7	1.1262	0.2243	1.1603	0.089*	
C8	1.0740 (4)	0.3116 (2)	1.0490 (3)	0.0556 (9)	
C9	1.1271 (4)	0.3676 (2)	1.0047 (3)	0.0562 (9)	
C10	0.8121 (4)	0.4607 (2)	0.8015 (3)	0.0523 (9)	
C11	0.8492 (4)	0.5283 (2)	0.7693 (3)	0.0617 (10)	
H11	0.9360	0.5443	0.8056	0.074*	
C12	0.7610 (4)	0.5723 (2)	0.6852 (3)	0.0729 (12)	
H12	0.7884	0.6172	0.6644	0.088*	
C13	0.6320 (4)	0.5498 (3)	0.6320 (3)	0.0755 (12)	
H13	0.5724	0.5788	0.5738	0.091*	
C14	0.5918 (5)	0.4853 (3)	0.6642 (4)	0.0812 (13)	
H14	0.5039	0.4711	0.6297	0.097*	
C15	0.6803 (4)	0.4402 (2)	0.7479 (3)	0.0678 (10)	
H15	0.6515	0.3958	0.7686	0.081*	

### Atomic displacement parameters (Å^2^)

**Table:**

	*U*^11^	*U*^22^	*U*^33^	*U*^12^	*U*^13^	*U*^23^
Se1	0.0824 (3)	0.0453 (3)	0.0779 (3)	−0.0142 (2)	0.0420 (2)	0.00255 (19)
N1	0.0679 (19)	0.0398 (17)	0.0617 (18)	−0.0123 (15)	0.0370 (16)	−0.0059 (15)
C1	0.071 (3)	0.041 (2)	0.060 (2)	−0.0144 (19)	0.036 (2)	−0.0107 (19)
C2	0.066 (2)	0.040 (2)	0.058 (2)	−0.0114 (17)	0.0373 (18)	−0.0113 (17)
C3	0.073 (3)	0.055 (2)	0.059 (2)	−0.0138 (19)	0.038 (2)	−0.0005 (19)
C4	0.068 (3)	0.085 (3)	0.066 (3)	−0.014 (2)	0.028 (2)	0.003 (2)
C5	0.070 (3)	0.107 (4)	0.068 (3)	−0.009 (3)	0.023 (2)	−0.003 (3)
C6	0.084 (3)	0.083 (3)	0.064 (3)	0.000 (3)	0.018 (2)	0.004 (2)
C7	0.089 (3)	0.063 (3)	0.073 (3)	−0.011 (2)	0.036 (3)	0.002 (2)
C8	0.074 (3)	0.047 (2)	0.053 (2)	−0.0104 (19)	0.033 (2)	−0.0098 (18)
C9	0.067 (2)	0.056 (2)	0.051 (2)	−0.0148 (19)	0.0311 (19)	−0.0088 (18)
C10	0.069 (2)	0.044 (2)	0.057 (2)	−0.0042 (18)	0.040 (2)	−0.0073 (17)
C11	0.074 (3)	0.052 (2)	0.076 (3)	−0.0048 (19)	0.048 (2)	−0.003 (2)
C12	0.099 (3)	0.055 (3)	0.084 (3)	0.007 (2)	0.058 (3)	0.012 (2)
C13	0.083 (3)	0.079 (3)	0.069 (3)	0.015 (3)	0.036 (3)	0.005 (2)
C14	0.077 (3)	0.087 (4)	0.074 (3)	−0.007 (3)	0.027 (2)	−0.001 (3)
C15	0.073 (3)	0.059 (3)	0.074 (3)	−0.012 (2)	0.034 (2)	−0.006 (2)

### Geometric parameters (Å, °)

**Table:**

Se1—C1	1.928 (4)	C6—H6	0.9300
Se1—Se1^i^	2.3439 (9)	C7—C8	1.408 (5)
N1—C1	1.301 (4)	C7—H7	0.9300
N1—C2	1.370 (4)	C8—C9	1.402 (5)
C1—C8	1.422 (5)	C10—C11	1.383 (5)
C2—C3	1.368 (5)	C10—C15	1.392 (5)
C2—C10	1.478 (5)	C11—C12	1.373 (5)
C3—C9	1.410 (5)	C11—H11	0.9300
C3—H3	0.9300	C12—C13	1.375 (5)
C4—C5	1.356 (6)	C12—H12	0.9300
C4—C9	1.415 (5)	C13—C14	1.354 (6)
C4—H4	0.9300	C13—H13	0.9300
C5—C6	1.380 (6)	C14—C15	1.381 (5)
C5—H5	0.9300	C14—H14	0.9300
C6—C7	1.354 (6)	C15—H15	0.9300
			
C1—Se1—Se1^i^	92.40 (12)	C9—C8—C1	116.1 (3)
C1—N1—C2	119.0 (3)	C7—C8—C1	125.2 (4)
N1—C1—C8	124.9 (3)	C8—C9—C3	118.6 (3)
N1—C1—Se1	117.5 (3)	C8—C9—C4	118.7 (4)
C8—C1—Se1	117.6 (3)	C3—C9—C4	122.7 (4)
C3—C2—N1	120.8 (3)	C11—C10—C15	117.3 (4)
C3—C2—C10	123.1 (3)	C11—C10—C2	122.0 (3)
N1—C2—C10	116.0 (3)	C15—C10—C2	120.7 (3)
C2—C3—C9	120.6 (3)	C12—C11—C10	121.6 (4)
C2—C3—H3	119.7	C12—C11—H11	119.2
C9—C3—H3	119.7	C10—C11—H11	119.2
C5—C4—C9	120.5 (4)	C11—C12—C13	119.8 (4)
C5—C4—H4	119.7	C11—C12—H12	120.1
C9—C4—H4	119.7	C13—C12—H12	120.1
C4—C5—C6	120.7 (4)	C14—C13—C12	120.0 (4)
C4—C5—H5	119.7	C14—C13—H13	120.0
C6—C5—H5	119.7	C12—C13—H13	120.0
C7—C6—C5	120.4 (4)	C13—C14—C15	120.6 (4)
C7—C6—H6	119.8	C13—C14—H14	119.7
C5—C6—H6	119.8	C15—C14—H14	119.7
C6—C7—C8	121.0 (4)	C14—C15—C10	120.7 (4)
C6—C7—H7	119.5	C14—C15—H15	119.7
C8—C7—H7	119.5	C10—C15—H15	119.7
C9—C8—C7	118.7 (4)		
			
C2—N1—C1—C8	−0.6 (5)	C7—C8—C9—C4	0.1 (5)
C2—N1—C1—Se1	−179.3 (2)	C1—C8—C9—C4	−178.8 (3)
Se1^i^—Se1—C1—N1	0.9 (3)	C2—C3—C9—C8	−0.1 (5)
Se1^i^—Se1—C1—C8	−177.9 (3)	C2—C3—C9—C4	179.9 (3)
C1—N1—C2—C3	1.8 (5)	C5—C4—C9—C8	0.3 (6)
C1—N1—C2—C10	179.4 (3)	C5—C4—C9—C3	−179.7 (4)
N1—C2—C3—C9	−1.5 (5)	C3—C2—C10—C11	−16.2 (5)
C10—C2—C3—C9	−178.9 (3)	N1—C2—C10—C11	166.3 (3)
C9—C4—C5—C6	−1.3 (7)	C3—C2—C10—C15	163.0 (3)
C4—C5—C6—C7	1.9 (7)	N1—C2—C10—C15	−14.5 (5)
C5—C6—C7—C8	−1.5 (7)	C15—C10—C11—C12	−2.3 (5)
C6—C7—C8—C9	0.4 (6)	C2—C10—C11—C12	176.9 (3)
C6—C7—C8—C1	179.2 (4)	C10—C11—C12—C13	0.9 (6)
N1—C1—C8—C9	−0.9 (5)	C11—C12—C13—C14	1.5 (6)
Se1—C1—C8—C9	177.8 (2)	C12—C13—C14—C15	−2.2 (7)
N1—C1—C8—C7	−179.7 (3)	C13—C14—C15—C10	0.7 (6)
Se1—C1—C8—C7	−1.1 (5)	C11—C10—C15—C14	1.6 (6)
C7—C8—C9—C3	−179.9 (3)	C2—C10—C15—C14	−177.7 (3)
C1—C8—C9—C3	1.2 (5)		

Symmetry codes: (i) −*x*+3/2, −*y*+1/2, −*z*+2.
